# Neural Tuning Functions Underlie Both Generalization and Interference

**DOI:** 10.1371/journal.pone.0131268

**Published:** 2015-06-25

**Authors:** Ian S. Howard, David W. Franklin

**Affiliations:** 1 Centre for Robotics and Neural Systems, School of Computing, Electronics and Mathematics, University of Plymouth, Plymouth, United Kingdom; 2 Computational and Biological Learning Laboratory, Department of Engineering, University of Cambridge, Cambridge, United Kingdom; The University of Western Ontario, CANADA

## Abstract

In sports, the role of backswing is considered critical for generating a good shot, even though it plays no direct role in hitting the ball. We recently demonstrated the scientific basis of this phenomenon by showing that immediate past movement affects the learning and recall of motor memories. This effect occurred regardless of whether the past contextual movement was performed actively, passively, or shown visually. In force field studies, it has been shown that motor memories generalize locally and that the level of compensation decays as a function of movement angle away from the trained movement. Here we examine if the contextual effect of past movement exhibits similar patterns of generalization and whether it can explain behavior seen in interference studies. Using a single force-field learning task, the directional tuning curves of both the prior contextual movement and the subsequent force field adaptive movements were measured. The adaptation movement direction showed strong directional tuning, decaying to zero by 90° relative to the training direction. The contextual movement direction exhibited a similar directional tuning, although the effect was always above 60%. We then investigated the directional tuning of the passive contextual movement using interference tasks, where the contextual movements that uniquely specified the force field direction were separated by ±15° or ±45°. Both groups showed a pronounced tuning effect, which could be well explained by the directional tuning functions for single force fields. Our results show that contextual effect of past movement influences predictive force compensation, even when adaptation does not require contextual information. However, when such past movement contextual information is crucial to the task, such as in an interference study, it plays a strong role in motor memory learning and recall. This work demonstrates that similar tuning responses underlie both generalization of movement direction during dynamic learning and contextual movements in both single force field and interference tasks.

## Introduction

When learning a new motor skill, such as tennis, it is necessary to develop an ability to perform movements using a racquet, including compensating for its dynamic properties in order to hit the ball appropriately. Furthermore, different strokes must be learned, refined by practice and stored in motor memory. During the game, the appropriate stroke must then be recalled and executed.

Humans adapt to changes in dynamics by forming a motor memory (or internal model) of the dynamics, which can then be used to compensate for the dynamics in a predictive manner [1–3]. This motor memory is gradually formed through trial and error, with the change in the predictive compensation scaling with the size of the error [4]. In order to investigate motor learning, robotic devices are typically involved in examining how participants compensate for novel dynamics applied to their hand during movement. Specifically, viscous force fields are often chosen for such studies since they have long lasting effects and are subject to interference [5,6].

If movements are performed within consistent novel environmental dynamics, such as a curl-field, rapid learning of the predictive controller takes place, resulting in a correspondingly rapid reduction in error. On the other hand, when movements are performed in opposing dynamics (such as curl-fields with opposing directions) which alternate or randomly switch from one movement to the next, substantial motor interference occurs and neither environmental dynamic is learned [5,7–11]. It has been shown that this interference can be reduced by means of both selective sensory cues and differences in the physical or visual state of the limb during the adaptation movement [11–17]. In addition, we have also previously shown that distinct past movements, each associated to different dynamics and occurring immediately prior to the adaptive movement, exhibit a strong contextual effect allowing the simultaneous learning of opposing force fields [18], This contextual effect decays rapidly with the time, producing no contextual effect if the two movements are separated by over one second. However when this past contextual movement takes place in the immediate past, it is equally effective at reducing the interference irrespective of whether it is actively generated, a passive contextual movement or is simply produced visually [18].

Although it is known that a motor memory may be learned in one particular limb configuration or for one or several specific movements, many studies have shown that such learned compensation is not only used locally. Rather it generalizes across the workspace and to other similar movements, although the effect of learning falls-off as movement deviates from the training direction. Initial studies suggested that the adaptation is learned and generalized relative to one’s own arm joint angles [1,19]. This view has been challenged by more recent studies suggesting that learning occurs in a mixed coordinate representation either with or without decay away from the local area of learning [20]. This theory that the learned compensation decays away from the learned state space is also consistent with other studies showing that learning is local [7,21,22]. Using careful analyses of the learned forces and the reduction in error during learning of a force field in eight equally spaced directions, it has been shown that the learning for any one movement affects movement in other directions [23,24]. This effect has been explained by hypothesizing that motor memory consists of a set of broadly tuned neural basis functions [23–26]. As such, although a movement made in one direction would mainly be affected by its closest basis functions, their effect would decay as movement direction changed.

In order to further examine the contextual effect of past movement, here we compared their generalization properties against those known to affect dynamic learning of viscous curl-fields. In particular we focus on the observation that there is a transfer of learning away from the trained movement direction that falls off as a function of angle [24]. We hypothesized that the contextual movement would exhibit a similar angular dependency on movement direction. To test this prediction, we first investigate parametric angular tuning of both contextual and adaptive movements in learning a single curl field. We then investigate the angular tuning of contextual movements in an interference paradigm, which involves learning adaptations to movements in pseudo randomly applied CW and CCW curl fields.

## Methods

A total of 20 right-handed participants (13 female; age = 20.5±3.3 means years) took part in two experiments. Participants provided written informed consent and were naïve to the aims of the experiments. The University of Plymouth Faculty of Science and Technology Human Ethics Committee approved the protocol and all participants were right handed based on the Edinburgh handedness questionnaire [27].

### Apparatus

Experiments were performed using a vBOT planar robotic manipulandum and associated virtual reality system [28]. The vBOT is a custom built back-drivable planar robotic manipulandum, which exhibits low mass at its handle. Handle position is measured using optical encoders sampled at 1000 Hz, and torque motors allow end-point forces to be specified. The position signal is used unfiltered, whereas velocity is computed by fitting a quadratic equation of motion, assuming constant acceleration, over a window that consisted of the 30 most recent position samples and associated time stamps. The vBOT is equipped with a force transducer (Nano 25; ATI) mounted at the handle to measure the applied forces. Prior to digitization, the output channels of the force transducers are low-pass filtered at 500 Hz using analogue 4th pole Bessel filters. Participants were seated in a sturdy chair in front of the apparatus and firmly strapped against the backrest with a four-point seatbelt to reduce body movement. Participants held the robot handle in their right hand while an air sled supported their right forearm on an air table, thereby constraining movement to the horizontal plane. Visual feedback was provided using a computer monitor mounted above the vBOT where the scene was projected veridically to the participant via a mirror, so the visual cursor seen by the participant appeared in the same plane and at the same location as their hand. Participants were prevented from viewing their hand directly, and instead the virtual reality system was used to overlay images of the starting location, via point, final target, (all 1.25 cm radius disks) and a hand cursor (0.5 cm radius red disk) in the plane of movement. In all experiments, data was collected from the manipulandum’s encoders and force transducer at 1000 Hz and logged to disk for offline analysis using Matlab (Matlab, The MathWorks Inc., Natick, MA, USA).

### Force Fields

In order to study the contextual tuning effect of past motion, participants performed reaching movements in which either a null field or a velocity-dependent viscous curl force field [7] was presented. In the curl force field condition, the force experienced at the manipulandum’s handle was given by:

$$\left[\begin{array}{c} F_{x}\\ F_{y} \end{array}\right]=k\left[\begin{array}{cc} 0 & -1\\ 1 & 0 \end{array}\right]\left[\begin{array}{c} \dot{x}\\ \ddot{y} \end{array}\right]$$

where *k* was set equal to ±13 N m^-1^ s. The sign of *k* determines the direction of the force-field (CW or CCW). In Experiment 1, each participant only experienced a single force field direction. In Experiment 2, each participant experienced both force field directions where the specific direction depended on the contextual phase of the trial.

In all experiments, the relationship between passive contextual movement and curl field direction (CW/CCW) was counterbalanced across participants to minimize any effect arising from the interaction of movement direction and curl field direction. The force-field adaptation was estimated by measuring kinematic error on field trials and force compensation on channel trials.

### Protocol

In the two experiments, participants always grasped the handle of the vBOT planar robotic manipulandum. On each trial the starting location, central location and final target were displayed and the vBOT then moved the participant’s hand to the start location (following a minimum jerk trajectory). Once the hand cursor remained within the start location at a speed below 0.1 cm/s for 500 ms, participants were cued by an acoustic tone indicating the start of the two-part reaching movement. The first part of the reaching movement was a passive contextual movement generated by the robotic manipulandum from the start to the central location. It was implemented by coupling a computer-generated movement following a minimum jerk trajectory of duration 640ms to the handle with a simulated spring with a stiffness coefficient of -20 N/cm. Once the hand reached the central location, participants immediately performed an active movement to the final target position. Participants were encouraged to perform the second movement immediately after the passive contextual movement reached the central position, and were required to do so within 250 ms or the trial was aborted (and then repeated). Moving to the final target before reaching the central position was not physically possible since the robot held the handle to the passive trajectory using the simulated spring. In practice participants generally remained at the central location for a short time, and the dwell time period for which they remained within the radius of the central location disc with a speed less than 5 cm/s was calculated.

The second movement constituted the adaptation movement, as it was only during this movement that force fields were introduced. If the duration of the adaptation movement (measured from the time the cursor had moved from the central location until it entered the target location) was between 150 and 300 ms, a “correct speed” message was displayed; otherwise the appropriate “too fast” or “too slow” warning was shown. Throughout each experiment participants were provided with a short rest break approximately every 200 trials (195–205 trials), although they could also take a break at any time by releasing the handle of the manipulandum.

### Experiment 1: Generalization of a single motor memory (n = 8)

**Fig 1:**
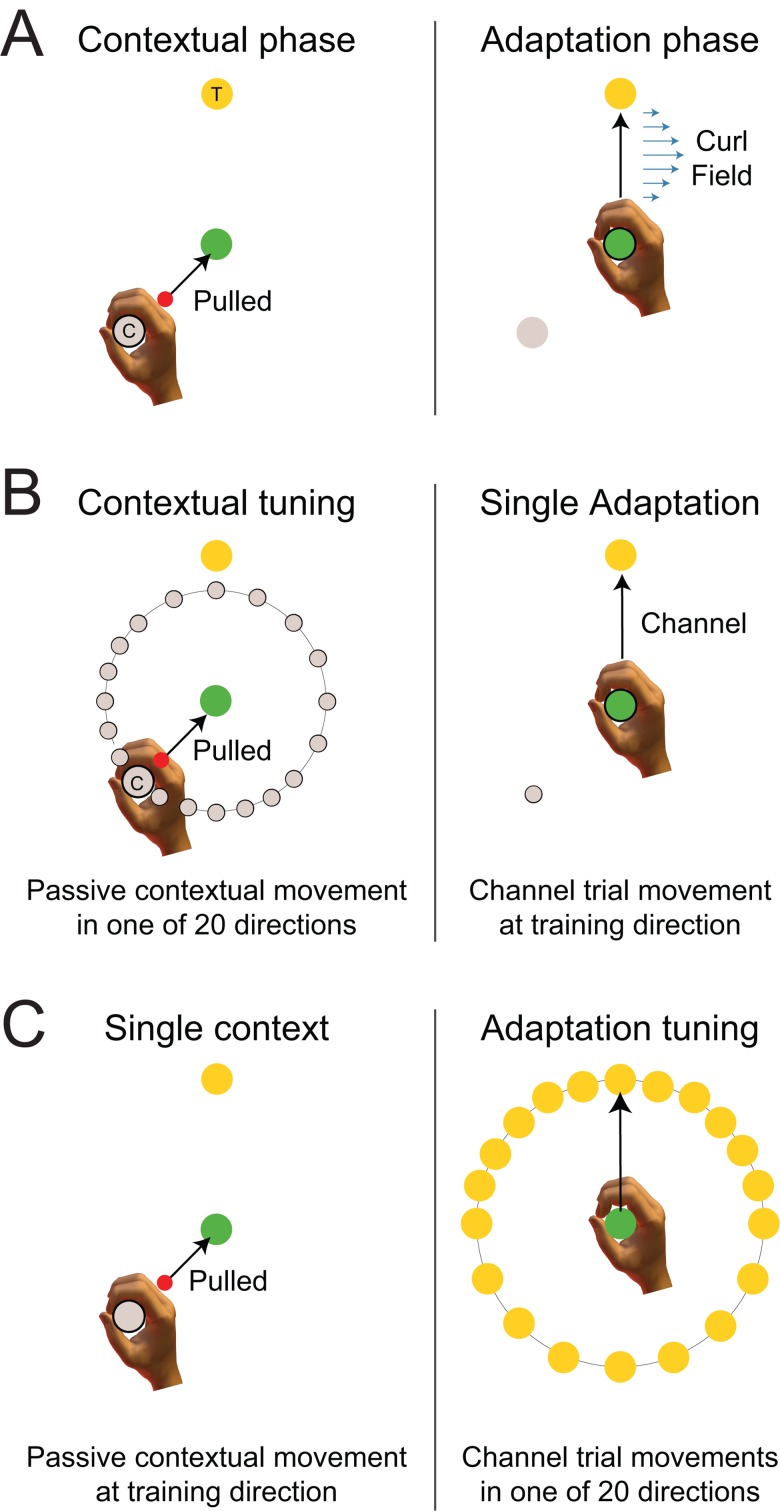
Single field tuning experimental paradigm. (A) On each trial, the participant’s hand was pulled to the start location (grey contextual target) by the robot. After the participant’s hand was within the start location for a fixed time, they experienced a passive contextual movement to a central location (green circle), termed here the contextual phase. Immediately after entering the central location, they were required to make an active movement to a final target location (yellow circle), termed the adaptation phase. During force field exposure, the participants were subject to a curl force field only within this adaptation phase. (B) In order to examine the generalization of the contextual movement direction, the adaptation movement target (yellow circle) location remained constant while the contextual movement starting point (grey circle) was varied. It was selected from one of the 20 starting points, concentrated around the contextual training direction, but spanning the full 360° range. On these trials, the adaptation movement was always made to a single target in a mechanical channel in order to measure the predictive force. (C) In order to examine the generalization effect of adaptation movement direction, the contextual movement training direction remained constant while the target locations (yellow circle) were varied. The final target location was selected from one of the 20 target locations concentrated around the adaptation movement training direction, and spanning the full range of 360°. The movement to the final target was always within a mechanical channel that was used to measure the predictive force.

The first experiment examined the directional tuning of contextual and adaptive movements using a single curl field-learning task, in which past motion context is not needed to learn compensation (Fig 1). Each trial consisted of a 2-part movement. On each trial, the participants’ hand was pulled to the start location by the robot. The start was located at +45° of the middle line towards the central via point; see Fig 1A left (or at -45°, depending on the counterbalance group). Participants then experienced a 10cm passive contextual movement to the central location. Immediately afterwards they were required to make a 12cm active adaptation movement from the central location to a final target located straight ahead at 0°, in which they were subject to a curl force field (Fig 1A right).

Throughout the experiment, to assess feed forward compensation to the curl force field, channel trials [29] were employed in which the contextual and adaptation movement directions were the same as during field training. In such a channel trial, the adaptation movement was confined to a simulated mechanical channel with a spring constant of 10,000 N/m [29,30]. The channel was only present during the second adaptation part of the movement.

To examine the generalization effect of contextual movement direction, the final target location remained fixed at the 0° target and the contextual movement starting point was varied. The latter was selected pseudo-randomly from 20 starting points, concentrated around the contextual training direction, but spanning the full possible 360° range (Fig 1B). Specifically the contextual movement deviated from training angle by 15° for the ±90° range, and then by 30° for the remaining full ±180° range. Again, the channel was only applied during the second adaptation part of the movement. The contextual movement generalization data was fitted using a parametric tuning curve.

To examine the generalization effect of adaptation movement direction, the contextual movement training direction remained fixed at the training location while the target locations varied. The latter was selected pseudo-randomly from 20 target locations concentrated around the adaptation movement training direction, again spanning the full possible range of 360° (Fig 1C). The adaptation movement direction deviated from the training angle by 15° for the range ±90°, and then by 30° for the remaining full ±180° range. All adaptation movements in the 20 directions were performed in a channel. The adaptation movement generalization data was similarly fitted using a parametric tuning curve.

Experiment 1 was run in six distinct phases and consisted of 1970 trials arranged as follows:

Pre-exposure: This was carried out to estimate baseline movement performance and enable participants to practice the task. This phase consisted of 6 blocks of 10 null trial blocks in which no forces were applied. A block consisted of 9 Null trials and 1 channel trial (Total 60 trials: 54 null trials, 6 training direction channel trials).

Pre-exposure Generalization Testing: This was used to ensure participants had prior experience of the wide range of generalization direction trials that would take place during the generalization phase of the experiment. This phase consisted of 2 sets of blocks containing 40 field and 40 tuning channel trials. In each block, every generalization condition was experienced pseudo-randomly within a channel. In total, there were 2 repetitions of each of the 40 channel trials. (Total 160 trials: 80 field trials, 80 generalization direction channel trials).

Pre-exposure: The null pre-exposure phase was repeated again before commencing the training. This consisted of 6 blocks of 10 trials in the null force field (Total 60 trials: 54 null trials, 6 training direction channel trials).

Exposure: During the exposure phase, the novel dynamics (curl force fields) were introduced. The phase consisted of 35 blocks of 10 field trial blocks (Total 350 trials: 315 field trials, 35 training direction channel trials).

Exposure Generalization Testing: Generalization of the learned predictive compensation was investigated by pseudo-randomly interspersing channel trials for a range of contextual and adaptation movement angles with the field exposure trials. This consisted of 10 sets of 128 trial blocks, which meant that there were 10 repetitions of each of the 40 channel trials. (Total 1280 trials: 880 field trials, 400 tuning direction channel trials).

Post-exposure: This was used to examine movement trajectories after the dynamic force fields were removed. This consisted of 6 blocks of 10 trial null trial blocks, in which no forces were applied. A block consisted of 9 Null trials and 1 channel trial (Total 60 trials: 54 null trials, 6 training direction channel trials).

### Experiment 2: Generalization and interference of two motor memories (n = 12)

**Fig 2:**
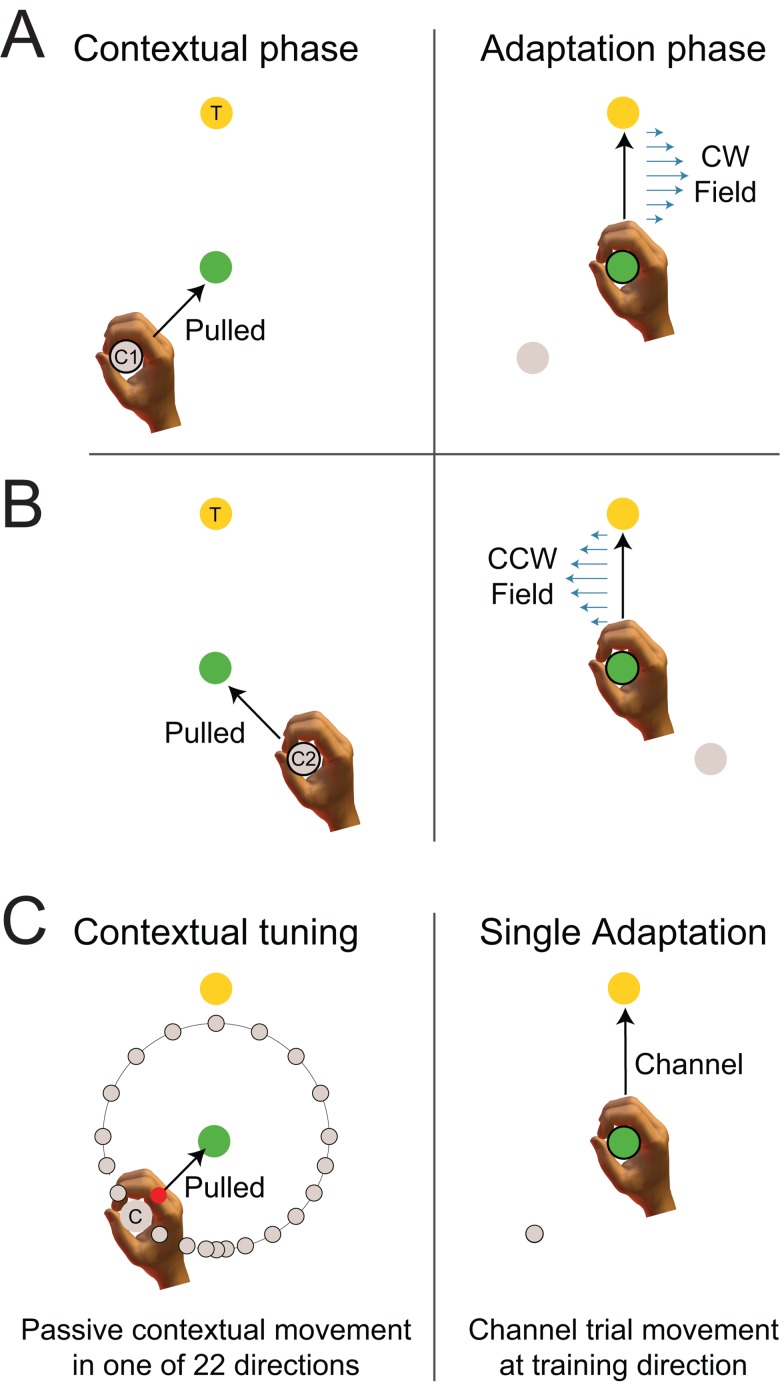
Interference tuning experimental paradigm. (A) On each trial, the participant’s hand was pulled to one of two possible start locations (grey circle) by the robot. Participants then experienced a passive contextual movement to a central location (green circle) and immediately afterwards were then required to make an active adaptation movement to a final target location (yellow circle). During force field exposure, participants were subject to a curl force field in this adaptation phase of the movement. The direction of the curl force field was consistently associated with one of the contextual movements, although the mapping was counterbalanced across participants. Here, the passive contextual movement from starting point C1 was associated with the clockwise (CW) curl force field. (B) The counter clockwise curl field (CCW) was associated with the passive contextual movement from starting point C2. Two groups of subjects performed the experiments. For one group the two contextual movements were located at ±45° as shown. The other group had contextual movements occurring from ±15°. (C) Mechanical channel trials were used in order to examine the affect of the contextual movement direction on the generalization of learning. The contextual movement starting point (grey circle) was varied from one of the 22 starting points, concentrated around the contextual training direction, but spanning the full range of 360°. The adaptation movement always occurred towards the training target location (yellow circle) and was implemented as a channel in order to measure the predictive force.

The second experiment investigated the directional tuning of the contextual effect of movement, using an interference task [18] in which past motion context was essential to learn compensation (Fig 2). Each trial again consisted of a 2-part movement, ending at either a target of 0° or one at 270°, chosen to be 90° apart to avoid interaction. One group of participants (n = 6) had contextual movements at ±45° relative to the direction of the adaptation movement, whereas a second group of participants (n = 6) had contextual movements at ±15°. For both groups, for each of the two targets the participants’ hand was first pulled to one of two possible start locations by the robot, located at either side of the middle line towards the central via point (Fig 2A & 2B left). Participants then experienced a 10cm passive contextual movement to the central location and immediately afterwards were required to make an active movement to the final target location (Fig 2A & 2B right). The first movement acted as a source of contextual information for the second movement, during which a randomly selected clockwise or counter-clockwise velocity-dependent curl field was applied that was associated with the contextual movement.

To assess learning of the opposing curl-fields throughout the entire experiment, channel trials were included for movements to the 0° target. One trial was preceded with the passive contextual movement associated with a CW field and the other trial was preceded with the passive contextual movement associated with a CCW field (randomizing which came first within each block).

As in Experiment 1, channel trials were also used to examine the generalization of learning, as the contextual movement starting point was varied while the adaptation movement target location remained fixed to the 0° target (Fig 2C, right). The 22 possible starting points were concentrated around the midpoint between the two contextual training directions spanning the full range of 360°. Specifically contextual movements started from points located at 180°, deviating by 5° for the first 2 data points, for 15° for the ±90° range, and then by 30° for the remaining full ±180° range (Fig 2C, left). The particular channel trial used on any given movement was selected pseudo-randomly.

The organization of trials in each instance of Experiment 2 was similar to the procedure adopted in Experiment 1. There were 1904 trials overall, organized as follows:

Pre-exposure: This consisted of 2 blocks of 40 null trial blocks, in which no forces were applied. A block consisted of 36 Null trials and 4 channel trials (Total 80 trials: 72 null trials, 8 training direction channel trials).

Pre-exposure generalization testing: This consisted of a block of 22 field trials and 22 tuning channel trials, in which all generalization conditions were experience pseudo-randomly within a channel (Total 44 trials: 22 field trials, 22 generalization direction channel trials).

Pre-exposure: This consisted of 2 blocks of 40 trials in the null field (Total 80 trials: 72 null trials, 8 training direction channel trials).

Exposure: This consisted of 15 blocks of 40 trials (Total 600 trials: 540 field trials, 60 training direction channel trials).

Exposure generalization testing: This consisted of 10 blocks of 102 trials of which 22 were generalization channel trials. (Total 1020 trials: 800 field trials, 220 tuning direction channel trials).

Post-exposure: This consisted of 2 sets of 40 trial null trial blocks, in which no forces were applied. A block consisted of 36 Null trials and 4 channel trials (Total 80 trials: 72 null trials, 8 training direction channel trials).

### Analysis

The data was analyzed offline using Matlab. To examine learning and generalization two measures were used: kinematic error on the adaptation movements and force compensation on the channel trials.

#### Kinematic error

The kinematic error was calculated on each adaptation movement as the maximum perpendicular error (MPE) of the hand path relative to a straight line joining the movement start to the center of the target location. For each participant, the MPE for all field exposure trials was averaged over 4 trials, with the sign flipped appropriately so that errors from CW and CCW field trials could be appropriately combined. The mean and standard error (SE) of MPE was then computed across all participants.

#### Force compensation

In order to look for evidence of feed forward adaptation, as opposed to relying on a reduction in kinematic error during force field learning which can also arise from muscle co-contraction [31–33], channel trials were randomly interspersed in each block of field exposure trials. Using this technique, measurement of endpoint forces at the handle onto the channel wall can estimate the amount of specific adaptation learned during field exposure [30,34]. More specifically, the force produced by participants perpendicularly into the wall of the simulated channel was first integrated across the adaptation phase movement. This integrated force was then divided by the value required to completely compensate for the field, as determined by the field strength and the corresponding integrated movement velocity on each trial. This yielded an estimate of the level of force compensation present as field exposure progressed [11,18]. The mean and SE force compensation was computed for all channel trials within each block across all the participants.

#### Modeling tuning response with a single Gaussian function

In the single curl field Experiment 1, the generalization channel trials correspond to both contextual and adaptation movement angles. The angular deviations from the training angle were first used to plot these two tuning functions. The counterbalanced dataset results were appropriately reflected so that all participants’ data in a given experiment could be combined into a single average tuning curve. The angular tuning curves for both contextual and adaptation movement had a single peak at the training direction, and the response fell off either side from this direction. We therefore fitted the Experiment 1 tuning response curves using the single Gaussian model function *M*_1_(*θ*):

$$M_{1}(\theta )=\frac{A}{\sigma \sqrt{2\pi }}\exp\left(-\frac{{\left(\theta -\mu \right)}^{2}}{2\sigma^{2}}\right)+B$$ (1)

where *θ* is the deviation angle from the training direction in degrees, and *μ* & *σ* are the mean and standard deviation of the Gaussian function respectively, A is a scaling factor and B is an offset.

#### Modeling interference response with the difference of two Gaussian functions

In the interference Experiment 2, the generalization channel trials corresponded to the contextual movement angles (namely either ±45° or ±15°). The tuning curves were bimodal, with peak magnitudes of opposite sign occurring near the training directions and passing through zero around the 0° movement directly between them. We modeled this response as the difference of two Gaussian functions having the same standard deviation, but opposite means and scaling factors, using the Gaussian difference model function *M*_2_(*θ*):

$$M_{2}(\theta )=\frac{A}{\sigma \sqrt{2\pi }}\left(\exp\left(-\frac{{\left(\theta -\mu \right)}^{2}}{2\sigma^{2}}\right)-\exp\left(-\frac{{\left(\theta +\mu \right)}^{2}}{2\sigma^{2}}\right)\right)$$ (2)

where *θ* is the deviation angle from the central direction in degrees, and *μ* & *σ* are the mean and standard deviation of the Gaussian function respectively, and A is a scaling factor. We note that the Gaussian offset factor B cancels out in this equation.

#### Fitting mean response data

The average compensation data across participants was fitted using non-linear optimization (Matlab function fmincon) that minimized the mean square error between the appropriate Gaussian model (Eqs 1 or 2) to the mean participant data. That is, the object function error E is the Euclidian distance between the model and response curves as given by:

$$E=\sqrt{\sum_{\theta }{\left(M(\theta )-R(\theta )\right)}^{2}}$$ (3)

Where θ is the contextual movement angle, R(θ) is the mean participant response at angle θ, and M(θ) is the model response at angle θ (using either Eq 1 for *M*_1_(*θ*) or Eq 2 for *M*_2_(*θ*) in Experiments 1 or 2 respectively).

To ensure a good fit was achieved on the single dataset in Experiment 1 and both datasets in Experiment 2, the optimization was run ten times for each case and the fit with the lowest error used.

#### Estimation of fitted response confidence limits

Bootstrap statistical analysis was performed to provide confidence intervals for the fitted response parameters. This was achieved by first randomly choosing participants with replacement, computing the average tuning curve over the chosen participants and then performing the parameter fitting (In Experiment 1 this involved the random selection of 8 subjects, and in Experiment 2 the random selection of 6 subjects). This procedure was repeated 1000 times and the results were used to estimate the mean and standard deviation of the fit.

## Results

The dwell time between the prior passive contextual movements and the adaptation movements in Experiment 1 was 106±5.4 ms (mean±SE). The dwell time between the prior passive movements and the adaptation movements were 114.2 ±4.14 ms and 111.2±2.74 ms for the ±45° and the ±15° conditions respectively.

### Experiment 1: Generalization of a single motor memory

**Fig 3:**
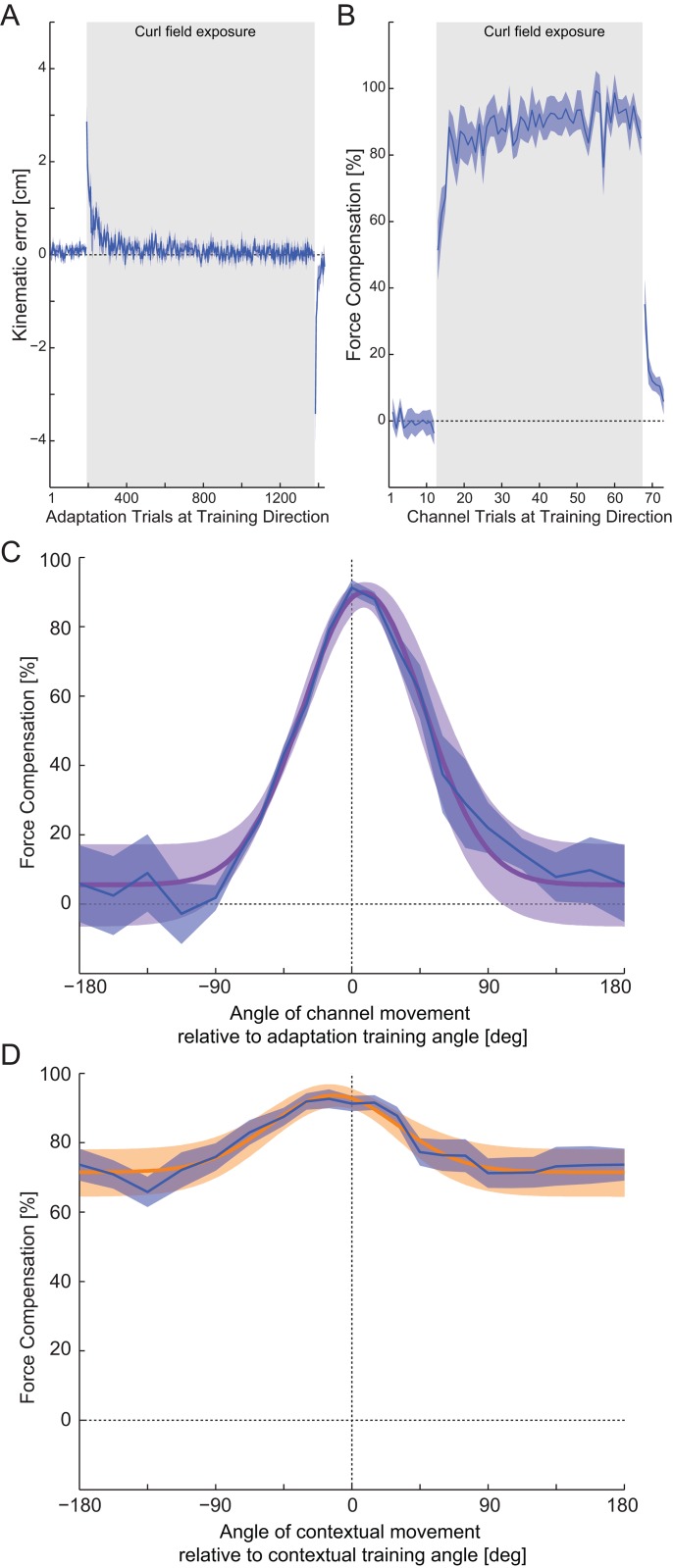
Single field tuning experimental results. (A) Mean kinematic error (solid line) and SE across participants (shaded region) during the adaptation movements across all blocks. The grey shaded region indicates the period over which the curl force field was applied. (B) Mean and SE of predictive force compensation for channel trials towards the training direction, expressed as a percentage of full compensation for the force field. (C) Tuning curves for the predictive force field compensation as the angle of the adaptation movement was varied, but the contextual movement remained at the training angle. The mean and SE of predictive force compensation (blue) are expressed as a percentage of full compensation. The experimental results were fitted with Gaussian tuning functions around the training direction. The mean and s.d. for the fitted Gaussian are plotted as a purple line and shaded purple region respectively. (D) The tuning curves (blue) and Gaussian fits (orange) for the predictive force field compensation as the contextual movement angle varied but the adaptation movement remained at the training direction.

Experiment 1 investigated the formation and generalization of a single motor memory as participants adapted to a single curl force field for one contextual movement and one adaptation movement. In the initial pre-exposure null condition phase, the movements did not deviate much from straight-line trajectories (Fig 3A). On the onset of during field exposure, there was a large increase in MPE, but then a rapid adaptation to the curl field such that final error levels (MPE) were close to those in the null field. After the field was removed in the post-exposure phase, large kinematic errors were again present (strong after effects) followed by a rapid reduction in MPE indicating a rapid washout of the learning. The rapid learning and adaptation was matched in the predictive force compensation data that was estimated at the training direction throughout the experiment (Fig 3B). During pre-exposure, the compensation was around zero. After onset of force field exposure, it rose rapidly to over 80%, indicating strong adaptation to the field. After field removal during post-exposure, the predictive force compensation rapidly decayed.

After the initial exposure, the generalization of the learned predictive force compensation was examined using channel trials in which either the contextual movement angle was varied or the adaptation movement angle was varied. The predictive force compensation was determined for each of these channels. The mean and SE across participants expressed as a percentage of full compensation is plotted as tuning curves for both adaptation (Fig 3C; blue curve) and contextual movements (Fig 3D; blue curve).

The adaptation angle tuning curve (Fig 3C) is similar to that seen by other researchers, showing a drop to around zero at ±90° angle [24]. The SE values increase outside this range, which arises due to the differences in behavior of participants to movements in the opposite direction to the training direction; some generalized by changing the sign of the adaptation whereas other participants did not.

#### Fitting the single field response

The adaptation tuning curve was fit with a Gaussian function (Eq 1) with a mean *μ* of 7.79°, a standard deviation *σ* of 39.97°, a scaling factor A of 0.0695 and an offset B of 0.00824 (Fig 3C, purple curve). The Gaussian function provided a good linear regression fit to the mean experimental data with an r^2^ value of 0.983, slope 0.982 and intercept 0.560%.

The contextual angle-tuning curve (Fig 3D; orange curve) shows similar characteristics to the adaptation angle-tuning curve, peaking at 0° with the same value and dropping to a minimum value away from the training angle. However this tuning curve is much shallower such that it never drops below 60%. This indicates that although there is a contextual effect of the past movement direction, the extent to which it can effect memory formation and recall in the subsequent movement is small when the participants learn a single force field. The contextual tuning curve was fit with a Gaussian with a mean *μ* of -12.07°, a standard deviation *σ* of 42.76°, a scaling factor A of 0.00798 and an offset B of 0.0434. The Gaussian provided a good linear regression fit to the mean experimental data with an r^2^ value of 0.938, slope 0.938 and intercept 4.89%.

#### Kolmogorov–Smirnov statistic

We used the KS statistic to examine how the mean participant tuning curves compared with their fitted Gaussian functions. The KS statistic is calculated over the ±180° range as the maximum absolute difference between the two respective cumulative distributions, computed by cumulatively summing their tuning curves and normalizing their overall sums to unity. Comparing the adaptation tuning curve with its fitted Gaussian yielded a KS value of 0.032. Comparing the contextual tuning curve with its fitted Gaussian yielded a KS value of 0.0037. Note that both values are well below the critical statistic value of the test (0.294) indicating that the Gaussian functions well fit the mean subject data.

### Experiment 2: Generalization and interference of two motor memories

Experiment 2 investigated the formation and generalization of motor memories as participants adapted to two opposing curl force fields during a single adaptation movement immediately preceded by one of two distinct contextual movements (each associated with one the two force field directions). Two groups of subjects performed this experiment. For one group the contextual movements were angled at ±45° relative to the adaptation movement (90° difference between the movements), whereas in the other group these movements were set at ±15° (30° difference between the movements).

**Fig 4:**
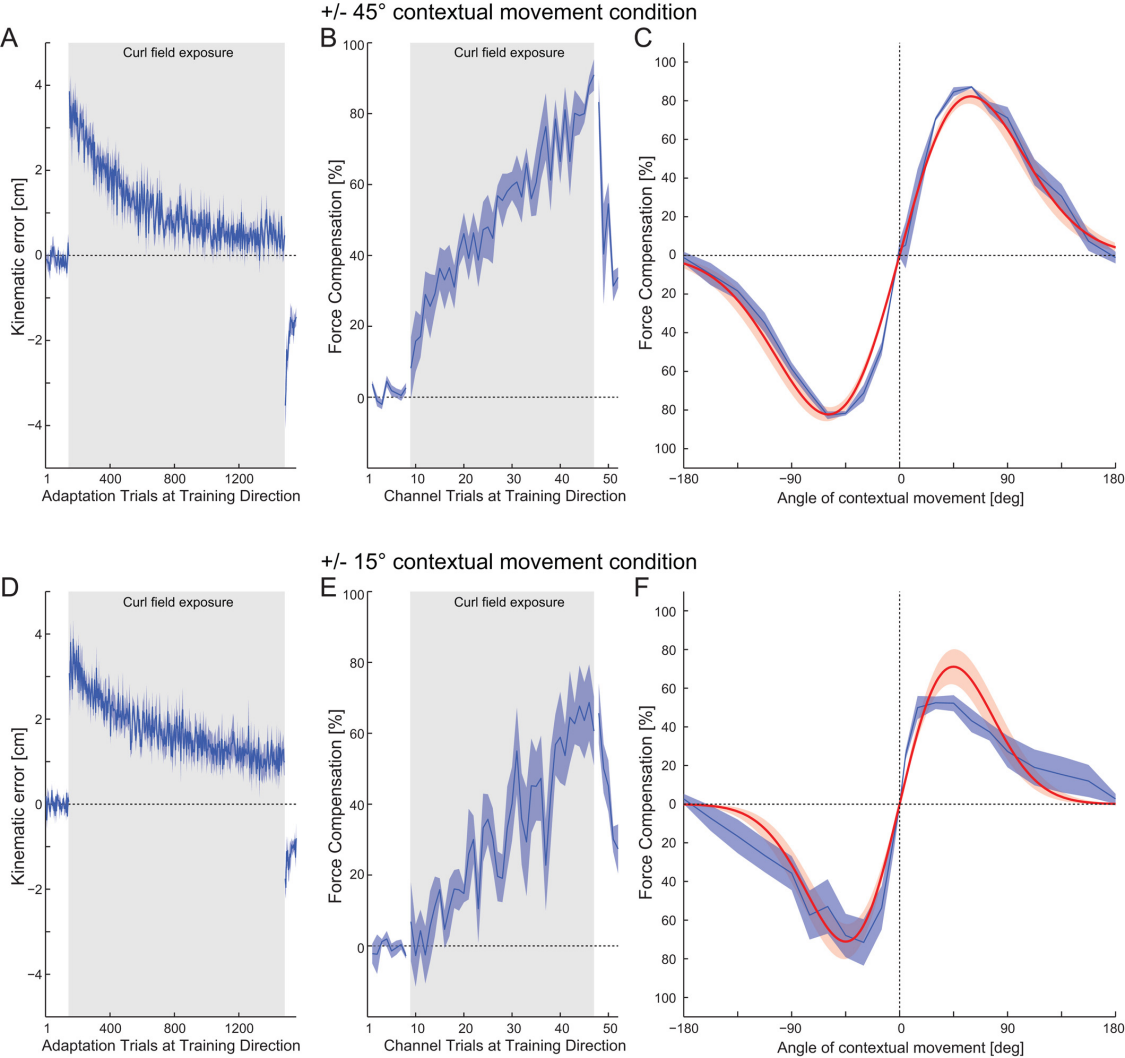
Interference tuning experimental results. (A) The mean kinematic error across all blocks (solid line) and SE across participants (shaded region) during the adaptation movements for the ±45° contextual movements. The grey shaded region indicates the period over which the curl force field was applied. (B) The mean and SE of predictive force compensation for the ±45° contextual movements, expressed as a percentage of full compensation for the force field. (C) The force compensation tuning curves (blue) plotted as a function of contextual movement direction for the ±45° contextual group. The mean and SE of predictive force compensation are expressed as a percentage of full compensation. The mean and s.d. of the best-fit Gaussian functions are shown in red. (D) The mean (and SE) kinematic error for the ±15° contextual movement group. (E) The mean (and SE) force compensation for the ±15° contextual movement group. (F) The force compensation tuning curves (blue) and the mean and s.d. of best-fit Gaussian functions (red) for the ±15**°** contextual movement group.

The participants in the ±45° movement context group showed significant learning from the initial to final exposure trials, with a large reduction in kinematic error (Fig 4A). This was paralleled by a significant increase in predictive force compensation during field exposure reaching up to 80% by the end of the exposure-learning phase (Fig 4B). However, at this point the ±15° group showed less learning both with a larger final level of kinematic error (F_1,550_ = 100.8; p<0.001) and a reduced final level of force compensation of about 70% (F_1,118_ = 32.073; p<0.001), see Fig 4D and 4E.

After the initial exposure, the generalization of the learned predictive force compensation was examined using channel trials in which the contextual movement angle was varied. The average percentage force compensation tuning curves across participants were plotted as a function of contextual movement direction (Fig 4C and 4F; blue curves). In both cases, a very pronounced tuning effect was observed, with compensation passing through zero at the critical point on the tuning curve around the mid-way between contextual movement training directions.

The larger learning effect seen with the ±45° versus the ±15° movement context group results suggest that in the latter condition, the tuning curves of the contextual movements may have exhibited more overlap. Consequently the ±15° contextual movements were less effective at partitioning the interference task into two motor memories, thus learning was slower, and overall less compensation was achieved by the end of the experiment. It can be seen that as the two training directions moved closer together (±45° to ±15°), the slope of the tuning curve between these two directions also became steeper, and compensation fell off more sharply as a function of movement direction.

#### Fitting interference response

We examined these tuning responses of the contextual movement in more detail by fitting a Gaussian difference function to the mean participant data (Eq 2). This function used the same single standard deviation, but opposite means and scaling factors for the two Gaussian components. The fits are shown as the red curves in Fig 4C & 4F. These linearly fit the mean experimental results well for both the ±45° (r^2^: 0.988, slope 0.988, intercept -1.50%) and ±15° (r^2^: 0.942, slope 0.942, intercept 2.59%) groups. The ±45° group was fit with a Gaussian with a mean *μ* of 39.25°, a standard deviation *σ* of 43.32°, a scaling factor A of 16.62 and an offset B of 0.0244. The ±15° group was fit with a Gaussian with a mean *μ* of 16.71°, a standard deviation *σ* of 43.32°, a scaling factor A of 15.16 and an offset B of 0.0275.

#### Predicting interference responses from single field responses

**Fig 5:**
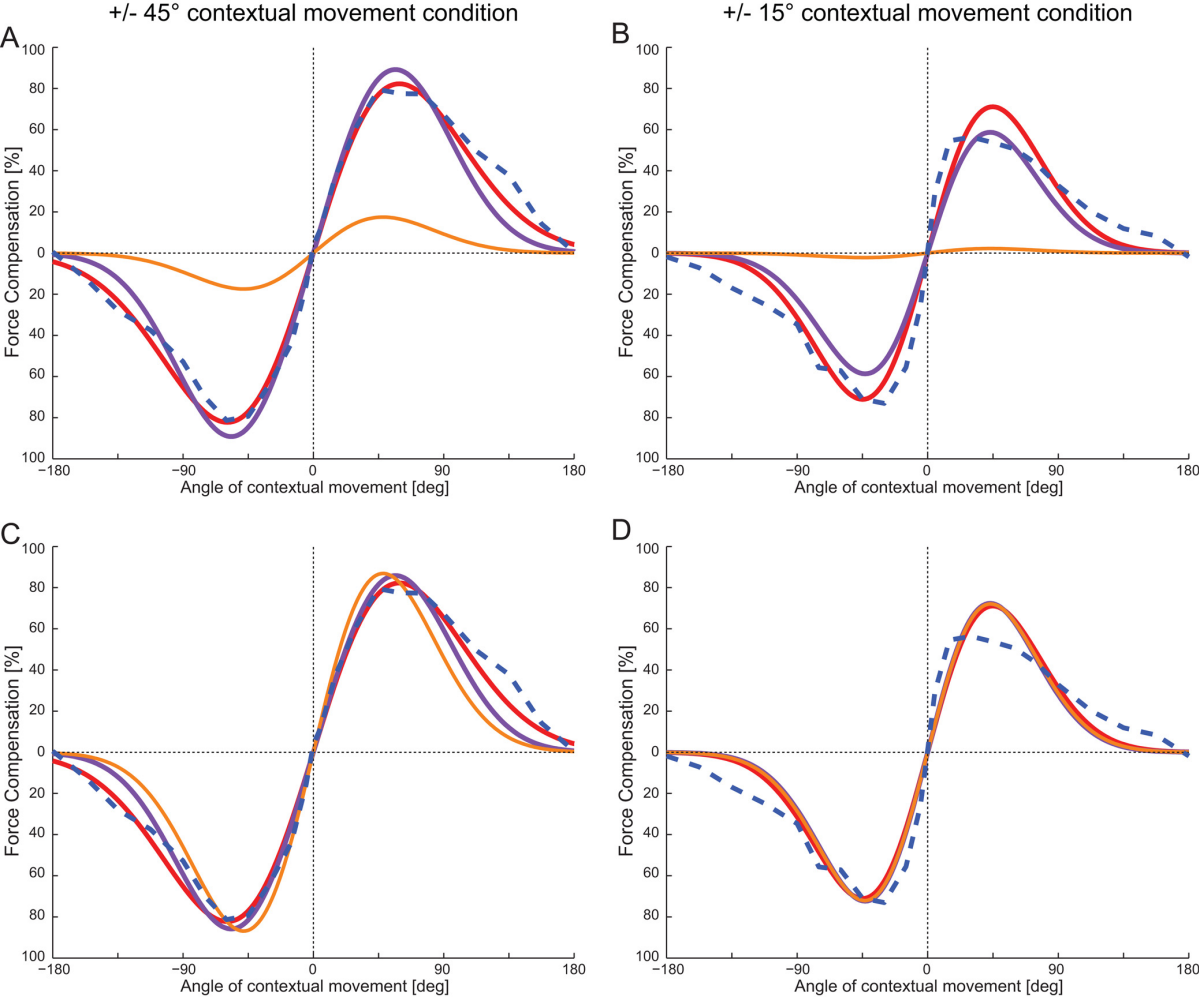
Interference responses can be predicted using tuning functions estimated from single a force-field condition. (A) The raw ±45° experimental results are shown in dashed blue. The ±45° interference response curve produced by the tuning function fitted to the ±45° interference data is shown in red. Overlaid are the predicted tuning functions estimated from the adaptation movements (purple) and passive contextual movements (orange) in the single field condition data. (B) The raw ±15° experimental results are shown in dashed blue. The ±15° interference response curve produced by the tuning function fitted to the ±15° interference data is shown in red. Overlaid are the predicted tuning functions estimated from the adaptation movements (purple) and passive contextual movements (orange) in the single field condition data. (C) Plotted as in A but linearly scaled to best fit the raw data. (D) As in B, again linearly scaled to best fit the raw data.

We examined to what extent the single field tuning responses from adaptation and contextual movements seen in Experiment 1 could explain the patterns of response generalization in interference Experiments 2. This was achieved by substituting the Gaussian model standard deviation and scaling parameters estimated in Experiment 1 into to Interference response Eq 2, with caveat that the mean values in the latter were set to the training locations of the interference experiments (namely ±45° and ±15° as appropriate). We note that the offset term cancels out in this computation and therefore plays no role in the estimate. The mean interference response curves were generated with the parameters estimated from either the contextual or adaptation movement responses. Results of the parameter substitutions are shown in Fig 5, which show model interference responses calculated on the basis of directly estimated parameters and substituted parameters, as well as the raw mean participant responses (dashed blue curves).

It can be seen (Fig 5A) that the absolute value of the response functions estimated directly from the ±45° interference data (red curve) and single force field adaptation data (purple curve) are in good agreement, whereas the single force field contextual data show a poorer fit (orange curve). Similar results are also seen for the ±15° interference data (Fig 5B). Here the responses estimated from the single force field adaptation tuning data (purple curve) are again in good agreement with the directly fitted data (red curve), whereas the predictions from the single force field contextual data do not.

The linear regression fit between the response estimated directly on the ±45° raw interference data and the prediction made from the static field adaptation tuning responses is good (r^2^ value of 0.987, achieved with a slope = 0.963 close to unity). The linear regression fit between the ±45° raw interference data and the prediction made from the static field contextual tuning responses also provided a good r^2^ value (0.944), but with a slope of 4.97 indicating a mismatch in scaling. Therefore by linear scaling the predicted interference plots using coefficients obtained from the regression analysis, good agreement between both predicted curves and the raw data was achieved (Fig 5C). Similarly good linear regression fits were also found for the ±15° raw data. Again the static field adaptation parameters provided a closer scaled fit than the static field contextual parameters (r^2^: 0.998, slope:1.23 and r^2^: 0.998, slope: 32.8 for the adaptation and contextual conditions respectively). Once more, scaled predicted interference plots also shown good agreement to the raw data (Fig 5D).

To summarize, comparing the tuning curves across the single force field and interference conditions demonstrates that the tuning characteristics of the adaptation movement generalization in the single force field condition could explain the directional tuning characteristics in the interference experiments. With appropriate scaling this was also the case for the contextual movement generalization in the single force field condition. It is important to note that we fitted average data across participants from the single field condition with Gaussian functions in order to explain responses seen in the interference experiments. We do not claim that the underlying neural tuning is specifically Gaussian, but rather that this model is a simplified fit to the data that predicts response in the interference experiment with minimal parameters. It can be seen that the widths of the Gaussians, which are used to model the underlying neural tuning functions, are well preserved across experimental conditions. This implies that mechanism that give rise to the tuning response seen in dynamic learning share similarities to the mechanism which leads to a response tuning effects in the passive contextual movement.

## Discussion

We tested the hypothesis that past passive contextual movement would exhibit an angular dependency on movement direction, as found previously for adaptation movements [23,24,26]. First we examined whether the contextual movement would exhibit angular tuning during the learning of a single curl force field. As expected the angular tuning curve for the adaptation movement peaked at 0° (trained direction) and exhibited generalization of the predictive learning away from the trained movement direction with a decay such that no predictive forces were present at ±90°. A directional tuning effect was also seen for the contextual movement directions. As the direction of the contextual movement varied, the tuning function showed similar characteristics to the adaptation angle-tuning curve, decaying as angle increased. However, this curve exhibited a large offset term so that the predictive force was always above 60% of force compensation. Therefore, although there was a contextual effect of the past movement direction, when only a single movement and force field were learned, its direct effect on the subsequent movement was limited.

In a second experiment, we investigated angular generalization behavior of two motor memories using an interference study. Participants learned to compensate for the two force fields using distinct prior contextual movements as in a previous study [18], this time in two groups of conditions; at ±45° and at ±15°. We found that learning was stronger for the group in which the contextual movements were further apart (±45° group). This result suggests that the tuning curves of the contextual movements in the ±15° group overlapped significantly. This hypothesis is also consistent with the observed width of the tuning curves found in the single field learning condition which indicated a standard deviation *σ* of around 43°, which indeed would lead to overlap when training directions were only 30° apart. We found that the single field contextual and adaptation tuning responses could be fitted with Gaussian functions. Moreover the estimated Gaussian parameters could explain the response curves seen in the interference experiments. This does not necessarily imply that the neural tuning functions are Gaussian, but rather the Gaussian fit to the average participant data constituted a good model that was able to explain the averaged response data.

Previous studies of the neural tuning functions that underlie the generalization of the learned predictive force to movements in different directions have investigated the error pattern from one movement to the subsequent movement [23,24]. The results of these studies show the gain (or transfer of learning) from the current movement to all other directions, or put another way, how much an error on the current movement effects other movement direction. These studies showed that the tuning function peaked at the current movement and decayed away to zero by 90°. Learning at movements in the opposite direction of current movement manifests itself differently depending on the form of the measure of error that is adopted. When the error measure is taken relative to an unchanging desired trajectory, the tuning gain shows a bimodal response, with a positive peak at 180° [23,24]. On the other hand, when the desired trajectory is allowed to vary, changing the estimated error measure, this bimodal response disappears and the tuning function is zero at 180° [24]. In our study, we do not need to consider the type of error measure used to determine generalization, as learning occurred only in a single movement direction while the tuning was examined using the predictive force on mechanical channels [29] in all movement directions. Using this technique we were able to directly measure the underlying generalization resulting from the neural tuning functions. Our mean results agree with those of Don chin and colleagues [24], suggesting that the desired trajectory of the movement may change as adaptation occurs [35]. However, in our adaptation movement data the variance of the compensation values increased towards the 180° direction, arising due to the differences in participant behavior. Some participants generated compensation such that its sign was opposite for reverse direction movements, whereas others generated compensation in the same direction. Thus, although the mean predictive force was around zero, each subject produced a predictive force against the channel wall with an absolute response of 42.14±4.76% (means) at 180°. Therefore our results do not directly support the suggestion that the underlying neural basis functions are not bimodal in form [24].

Our previous work has shown that prior movement allows the formation of independent motor memories [18]. This suggests that each prior contextual movement might have a neural tuning function associated with it. In this study we measured these tuning functions for a single force field learned at a single reaching movement. In this condition, there is no requirement for the prior movement to exhibit a contextual tuning function. However, these movements exhibited a tuned response, with the highest value at the learned contextual movement direction and decreasing with decay similar to that of the adaptation movement. However, unlike the adaptation movement, the predictive response was always above a compensation level of 60%. In the current study the dwell times for the prior movement were around 110 ms in all experiments. Our previous work has shown that the contextual effect of prior movement decreases dramatically with the length of the dwell time [18]. We would therefore expect that the contextual tuning function would be stronger (showing a larger decrease away from the learned direction) as the dwell time decreased towards zero. Conversely we would expect tuning to become flatter, and finally exhibit no tuning effect, as the dwell time increased towards 1000 ms, since at this dwell time contextual effects are known to disappear [18].

In the interference experiments a very pronounced tuning effect was observed, with compensation passing through zero at an angle midway between the two contextual movement training directions. As the two training directions moved closer together, the slope of the tuning curve between these two directions became steeper, and compensation fell off more sharply as a function of movement direction. From the single motor memory experiment, it was seen that the prior contextual movement exerted a tuning effect on the predictive commands, even when the task did not require its influence. Although this contextual tuning was modest (~20%) in the single field experiment, when the contextual effect was crucial to the motor learning task (as in the interference experiments) this was sufficient to partition the learning task into two separate motor memories, which were able to compensate for the two opposing curl fields.

The interference study demonstrated that subjects could learn to compensate for two opposing force fields for the same adaptive movement as long as the prior contextual movements were distinct, similar to results found in our previous study [18]. However, with a larger angular difference in the two contextual movements, the amount of adaptation was higher as indicated by both the reduction of error and the larger predictive force compensation for that group. We predict that the maximum reduction in interference will occur when the two contextual tuning functions exhibit no overlap, which, based on the contextual angular tuning results, would occur at ±90°. On the other hand, as the contextual angles become closer and closer we predict the interference will increase until the two force fields cannot be learned independently. We observed that in both the ±45° and ±15° interference study the peak location of the two Gaussian tuning functions were close to the training locations. In the latter case (±15°), the two opposite tuning functions will overlap and interfere with one another. Consequently the compensation force would increase as the testing contextual angle increases away from ±15°. The overlap of the two contextual tuning functions therefore explains why in this case the largest predictive force responses are found at angles that are greater than the trained directions (around ±30°).

The neural tuning of the contextual responses likely arises due to the directional tuning of neural activity that has been demonstrated throughout the visuo-motor system. For example, the neural activity in primary motor cortex is tuned to arm orientation [36] and movement direction [37]. More recently there has been fMRI evidence that directionally tuned neurons also present in the human visuomotor system [38]. Although the nature and implication of such directional encoding are by no means clear [39], such tuning is consistent with the observed of directional tuning effect of contextual movement. Indeed, these locally tuned responses have been associated with the formation of gain-fields that appear to underlie many behavioral results [15,16,40].

The neural basis for the contextual effect of immediate past movement on dynamic learning in the subsequent movement is still unclear. However we suggest that the sensory activity arising from the first contextual movement changes which neural units will be active in the second movement. Specifically, the observed phenomena suggest that distinct past movement states affect the motor system so that separate motor memories can be formed. In the single force field (Experiment 1) changes in contextual movement direction thereby resulted in a modulation of the recall of the dynamic compensation learned at the training direction, leading to the observed tuning effect. In the interference study (Experiment 2), the activity arising from distinct past movements enabled the formation of two separate motor memories instead of a single motor memory. Furthermore, changes in contextual movement direction again lead to the observed tuning effect.

There are many ways in which such recent states could affect motor learning. One proposed mechanism involves engaging separate populations of neurons [41,42]. Several recent multi-compartment models have been developed to mathematically formalize the context-dependent reduction in interference [43,44]. In our case, recent sensorimotor states, which might be represented by cerebellar output, may provide the signals that engage such different representations.

An alternative interpretation follows the work of Church land and colleagues, who regard the neural ensemble in, for example the primary motor cortex M1, as a dynamical system [45,46]. Within their framework, initiation or planning of movement leads to a transition of ensemble activity from a wandering state to a specific location in neural state space, in which it initializes the dynamical system to the state necessary to generate the required movement. Under this theory, our results would suggest that when distinct past movements precedes the adaptation movement, it uniquely affects the initial state of the dynamical system. Changes in the past contextual movement direction again modulate the recall of dynamics, providing both an explanation for the tuning curves and the observed separation in the representation of opposing dynamics for the interferences tasks.

Our previous work [18] has shown that past active and visual observation of movement invoke the same contextual effects as the past passive movement studied in this experiment. Similarly, these contextual effects can be invoked by changes in the physical or visual location of movement [11]. Thus it appears there are several ways in which contextual information can affect the motor system. Finally it was recently demonstrated that a contextual effect is also generated by future movement [47]. Since it is known that that future motor planning affects neural activity [48], it is possible that this follow-through effect may operate by affecting neural populations in a similar fashion to past movements.

Overall, our results demonstrate that the contextual effect of past movement influences motor learning, even when the task does not require this influence, such as the learning of a single curl field. Moreover, this prior movement effect exhibits an angular tuning characteristic, akin to that seen during the adaptation movement. Importantly, when the contextual effect of past movement is crucial to the dynamic learning task, as in the interference task, its effect plays a very strong role in motor memory learning and recall. Our results demonstrate that these generalization effects in both the contextual and adaptation movements arise through mechanisms that exhibit similar tuning, and may indicate a single neural mechanism underlying both effects.
